# Supplementary material for: Feasible supply of steel and cement within a carbon budget is likely to fall short of expected global demand
Source: Nat Commun. 2023 Nov 30;14:7895. doi: 10.1038/s41467-023-43684-3 (PMC10689810; doi:10.1038/s41467-023-43684-3)
Supplement: Supplementary file 1 — Supplementary Information [file 41467_2023_43684_MOESM1_ESM.pdf]

Supplementary Information

# Feasible supply of steel and cement within a carbon budget is likely to fall short of expected global demand

Takuma Watari [1,2], André Cabrera Serrenho [2], Lukas Gast [2], Jonathan Cullen [2], and Julian Allwood [2]

1. Material Cycles Division, National Institute for Environmental Studies, Japan
2. Department of Engineering, University of Cambridge, United Kingdom

Contact: Takuma Watari (watari.takuma@nies.go.jp)

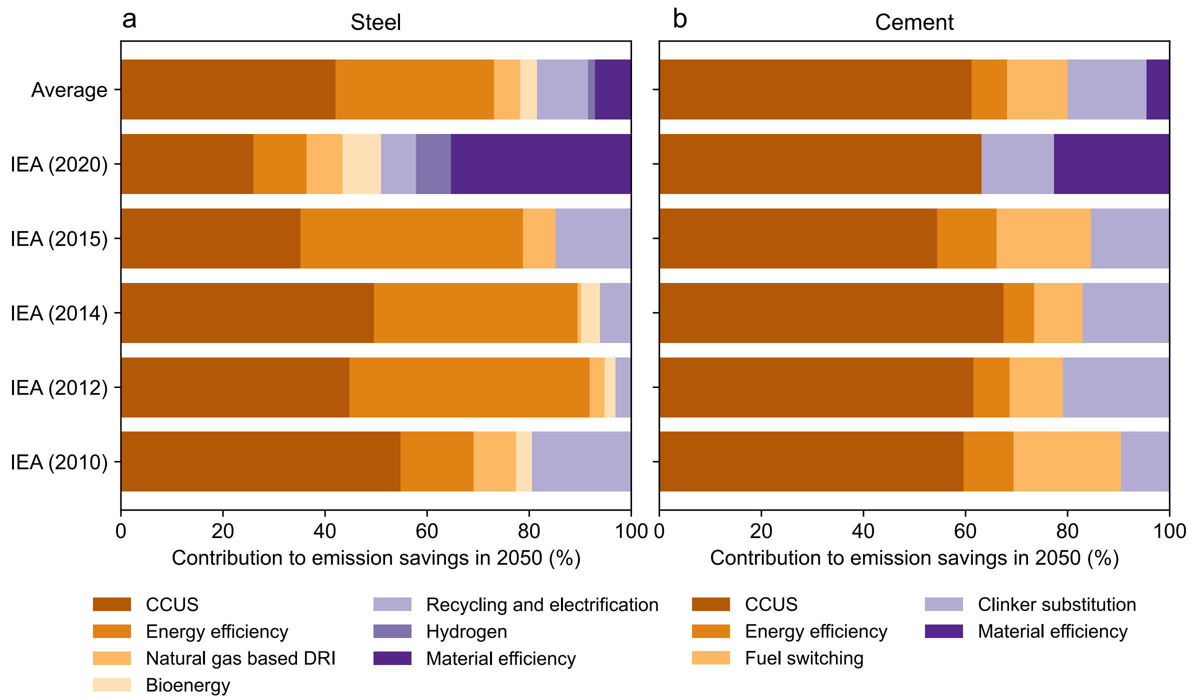


**Supplementary Fig. 1** Contributions of different strategies to 2050 emission savings in the steel and cement sectors. (a) Steel. (b) Cement. Emission savings are calculated as the difference between the baseline and mitigation scenarios (e.g., 6DS and 2DS). We examined all Energy Technology Perspectives reports [1–9] and obtained the available data from those reports. Some reports are therefore not included in this figure.

**Supplementary Table 1** Summary of system variables and parameters.

| Symbol | Description | Data |
|---|---|---|
| $P_i$ | Material production in route $i$ | Optimization variables |
| $\mathrm{CI}_i^{(f)}$ | Emission intensity of material production in route $i$ due to fuel combustion and chemical process. | Tables S2 and S3 |
| $\mathrm{EI}_i$ | Electricity intensity of material production in route $i$ | Tables S2 and S3 |
| $\mathrm{CI}^{(e)}$ | Emission intensity of electricity use | Fig. 1f in the main text |
| $\mathrm{CC}$ | Captured carbon through CCUS | Fig. 1c in the main text |
| $\mathrm{EI}_{\mathrm{cc}}$ | Energy penalty of carbon capture technologies | Table S4 |
| $\mathrm{Cap}_{\mathrm{Carbon}}$ | Carbon budget | Figure S3 and S4 |
| $\mathrm{Cap}_{\mathrm{Electricity}}$ | Maximum electricity supply | Fig. 1e in the main text |
| $\mathrm{Cap}_{\mathrm{F\_DRI}}$ | Maximum production via fossil DRI-EAF route | Figure S5 |
| $\mathrm{Cap}_{\mathrm{EAF}}$ | Maximum production via scrap-EAF route | Mass-balancing |
| $\mathrm{Pre}_{\mathrm{EAF}}$ | Recovered pre-consumer scrap for use in scrap-EAF route | Mass-balancing |
| $\mathrm{Post}$ | Recovered post-consumer scrap | Mass-balancing |
| $\theta$ | EAF production yield | 96% (Ref [10]) |
| $\delta$ | Forming yield | 93% (Ref [11]) |
| $\lambda$ | Fabrication yield | 86% (Ref [11]) |
| $\omega$ | Ratio of scrap in total BOF input | 20% (Ref [10]) |
| $\gamma$ | Recovery rate of post-consumer scrap | Figure S6 (calculated based on Refs [10,11]) |
| $\rho$ | Hibernation ratio of end-of-life products | 15% (Ref [12]) |
| $\phi$ | Lifetime distribution | Note |

Note: We assume a normal distribution with a mean of 38 years for steel and 50 years for cement and a standard deviation of 30% of the mean [13–15].

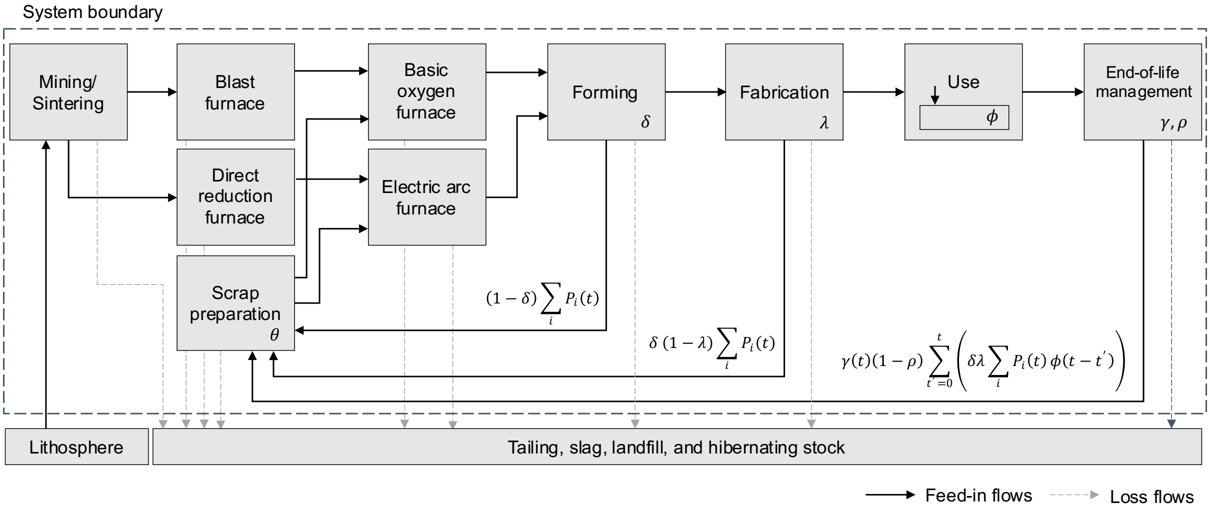


**Supplementary Fig. 2** Conceptual flow chart of iron and steel flows. The symbols at the bottom right of the process show the associated coefficients; the three equations show scrap estimation formulas based on simple mass balance. $\theta$: EAF production yield. $\delta$: Forming yield. $\lambda$: Fabrication yield. $\phi$: Lifetime distribution. $\gamma$: Recovery rate of post-consumer scrap. $\rho$: Hibernation ratio of end-of-life products.

**Supplementary Table 2** Key parameters that determine the emission intensity of crude steel production. Data are based on Refs [12,16–19]. Note that the data for the H2 DRI-EAF process include electricity use for green hydrogen production. The system is based on Ref [19] with an electrolyzer efficiency of 45 kWh/kg $H_2$ and a hydrogen mass flow rate of 1.5 (i.e., 50% oversupply of hydrogen for full conversion of iron ore in the shaft [20]).

| | Fuel combustion and chemical process (kg-CO2/t-steel) | Electricity use (kWh/t-steel) |
|---|---|---|
| BF-BOF | 1,996 | 328 |
| BF-BOF with top gas recycling and coke substitution | 1,680 | 305 |
| Fossil DRI-EAF | 840 | 117 |
| H2 DRI-EAF | 153 | 3,768 |
| Scrap-EAF | 100 | 508 |

**Supplementary Table 3** Key parameters that determine the emission intensity of cement production. Data are based on Refs [21–23].

| | Current | Target | Unit |
|---|---|---|---|
| Clinker-to-cement ratio | 72 | 57 | % |
| Thermal efficiency in the cement kiln | 3.4 | 3.0 | GJ/t-clinker |
| Milling/grinding electrical efficiency | 102 | 85 | kWh/t-cement |
| Carbon intensity of fuel mix | 85 | 58 | kg-$CO_2$/GJ |

**Supplementary Table 4** Overview of carbon capture technologies and energy penalties. Data are based on Refs [24,25]. Note that our simplified model does not take into account detailed processes within the plant (e.g., $CO_2$ purity and capture rates). Therefore, the model only considers the potential range of total carbon capture in the sector and the energy penalty to estimate the maximum global materials production within Paris-compliant carbon budgets.

| | Overview | Value |
|---|---|---|
| Steel sector | This study assumes the use of post-combustion with membranes due to the high technology readiness level [24] (*). The system can be effectively operated using electrical energy. | 1.8 GJ/t-$CO_2$ |
| Cement sector | This study assumes the use of membrane-assisted $CO_2$ liquefaction, which has the advantage of being easily retrofitted and requires only electricity as an input to the process [25]. | 3.2 GJ/t-$CO_2$ |

(*) It should be noted that many previous studies have assumed post-combustion with chemical absorption as the carbon capture technology in the steel sector [24]. Therefore, there is significant uncertainty in the energy penalty of post-combustion with membranes, which can be up to 4.4 GJ/$CO_2$. We explore the impact of this uncertainty in the form of a sensitivity analysis (**Supplementary Fig. 8**).

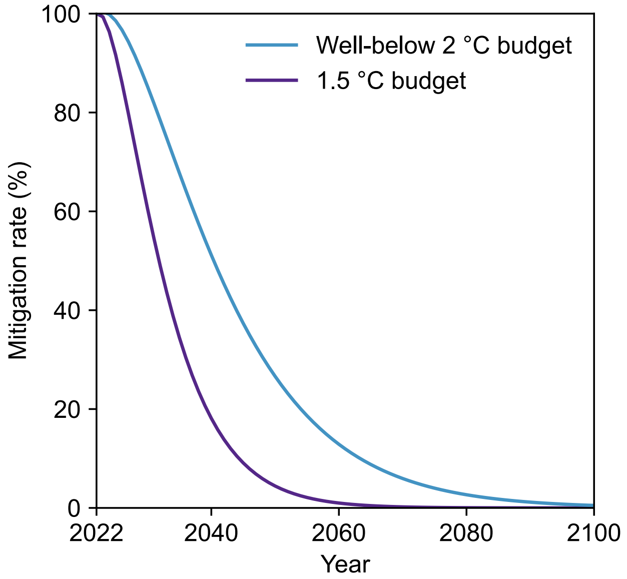


**Supplementary Fig. 3** Annual emissions mitigation rate for Paris-compliant carbon budgets. The carbon budget is based on limiting the global mean temperature rise within 1.5 °C with a 50% probability, consistent with the Paris Agreement pledges (~420 Gt-$CO_2$) [26]. We also consider a carbon budget for a 50% probability of 1.7°C (equivalent to an 83% probability of 2.0°C), which corresponds to “well below 2°C” (~770 Gt-$CO_2$) [27]. The annual emissions mitigation rate is determined using equation 3 in Ref [28].

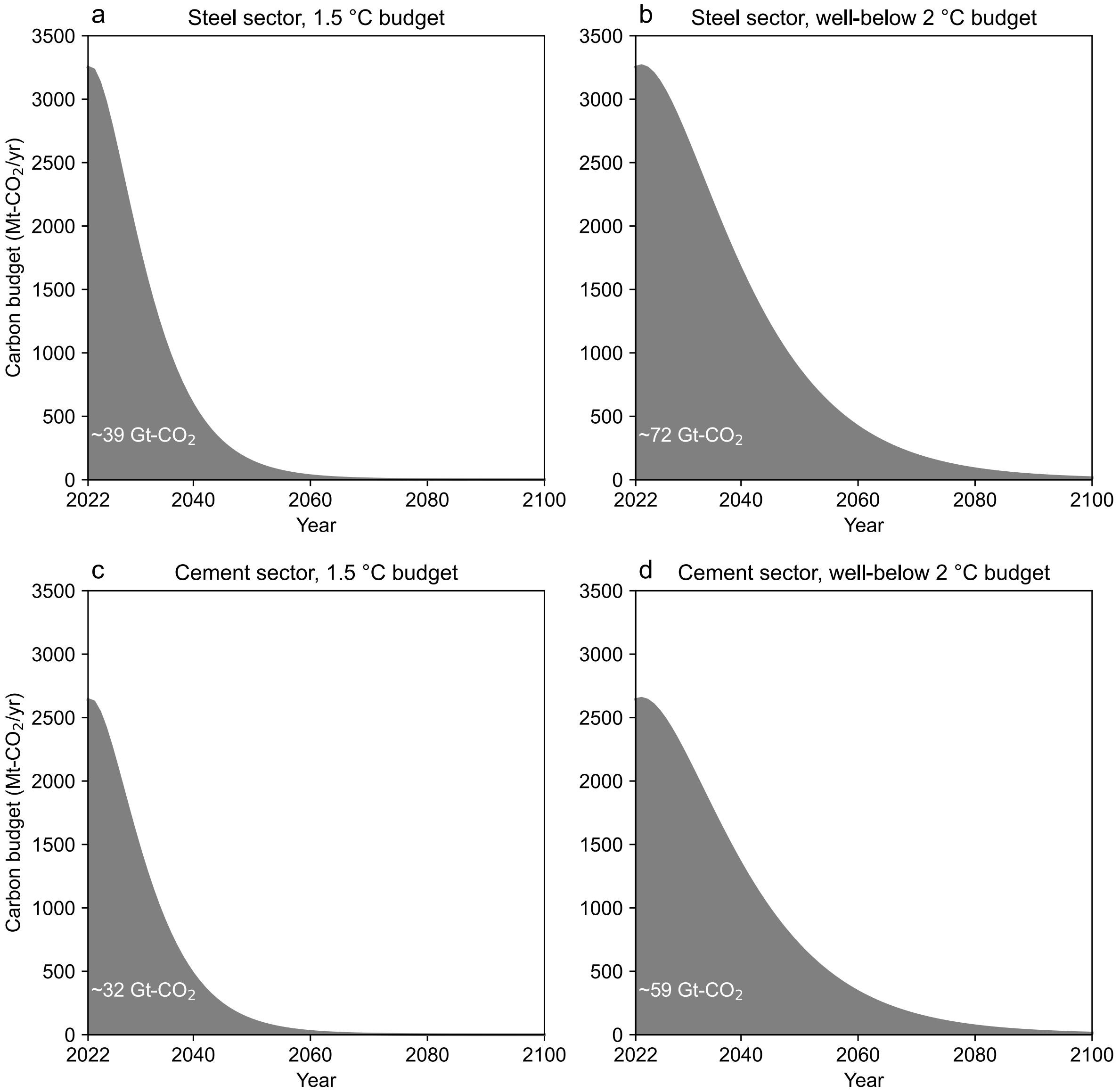


**Supplementary Fig. 4** Paris-compliant carbon budgets for the steel and cement sectors. (a) Steel sector, 1.5 °C budget. (b) Steel sector, well-below 2 °C budget. (c) Cement sector, 1.5 °C budget. (d) Cement sector, well-below 2 °C budget. We allocate the total carbon budgets to the global steel and cement sectors by multiplying the current emissions of the steel and cement sectors by the annual emissions mitigation rate shown in **Supplementary Fig. 3**.

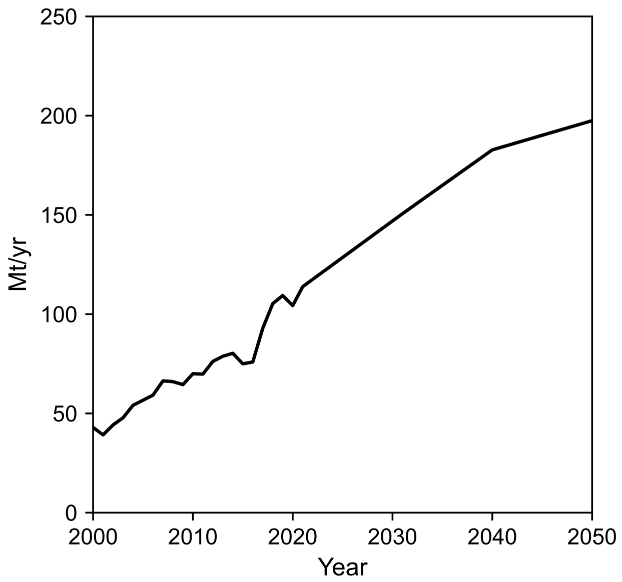


**Supplementary Fig. 5** Maximum deployment of fossil DRI-EAF route. Fossil fuel-based DRI production is strongly influenced by regional factors and has been predominantly deployed in the Middle East and India, where significant fossil fuel reserves exist [29]. In this study, the maximum deployment of fossil fuel-based DRI production is determined exogenously based on a detailed analysis conducted by the IEA [7]. The IEA's analysis takes into account various factors such as regional fossil fuel availability, technological feasibility, and environmental considerations to establish the upper limit for the deployment of this production technology.

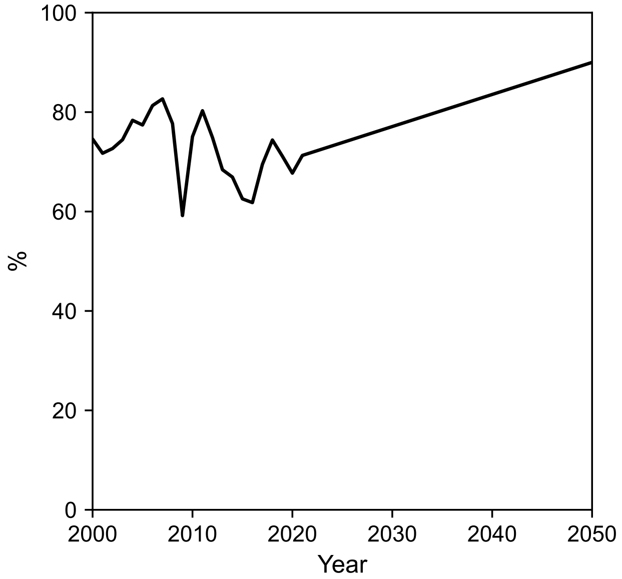


**Supplementary Fig. 6** Recovery rate of post-consumer scrap. The historical data are calculated based on Ref [11] and the 2050 target value is based on Ref [10].

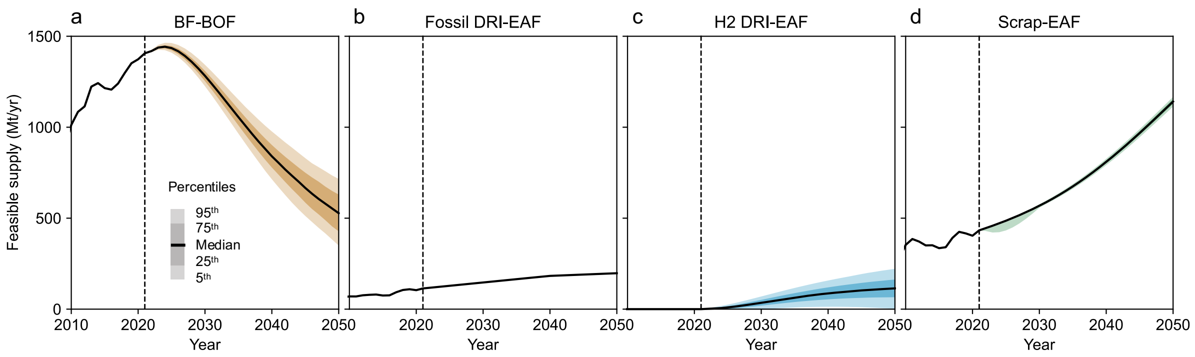


**Supplementary Fig. 7** Global crude steel production per process within a well-below 2 °C budget by 2050. (a) BF-BOF: blast furnace and basic oxygen furnace route. (b) Fossil DRI-EAF: fossil fuel-based direct reduced iron and electric arc furnace route. (c) H2 DRI-EAF: hydrogen-based direct reduced iron and electric arc furnace route. (d) Scrap-EAF: scrap-based electric arc furnace route.

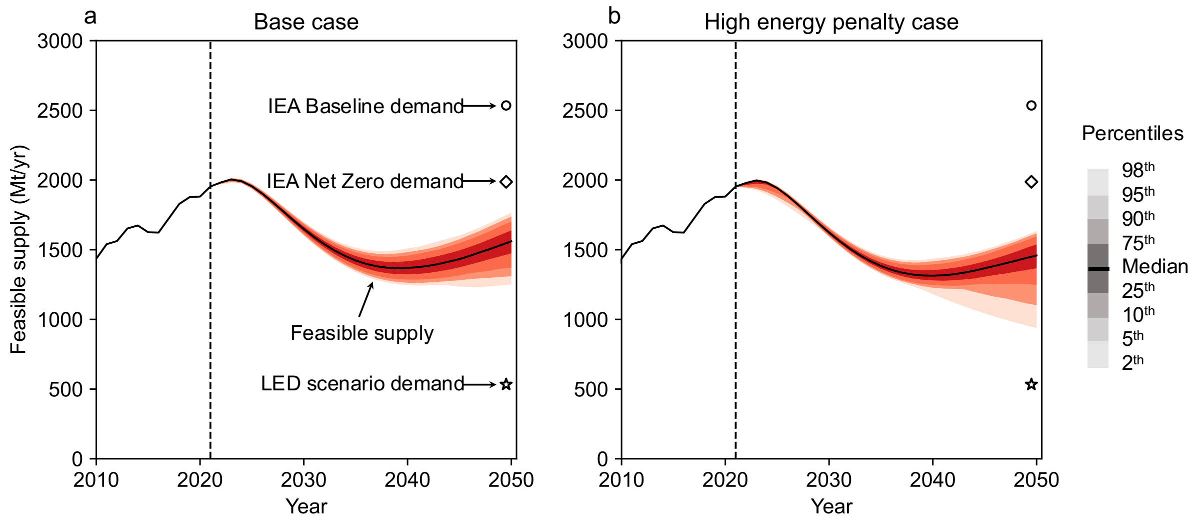


**Supplementary Fig. 8** Sensitivity of feasible steel supply within a 1.5 °C budget to the energy penalty of carbon capture technologies. (a) Base case: 1.8 GJ/t-$CO_2$. (b) High energy penalty case: 4.4 GJ/t-$CO_2$. The high energy penalty case has a wider range of feasible supply uncertainty and a median in 2050 that is about 7% lower than the base case.

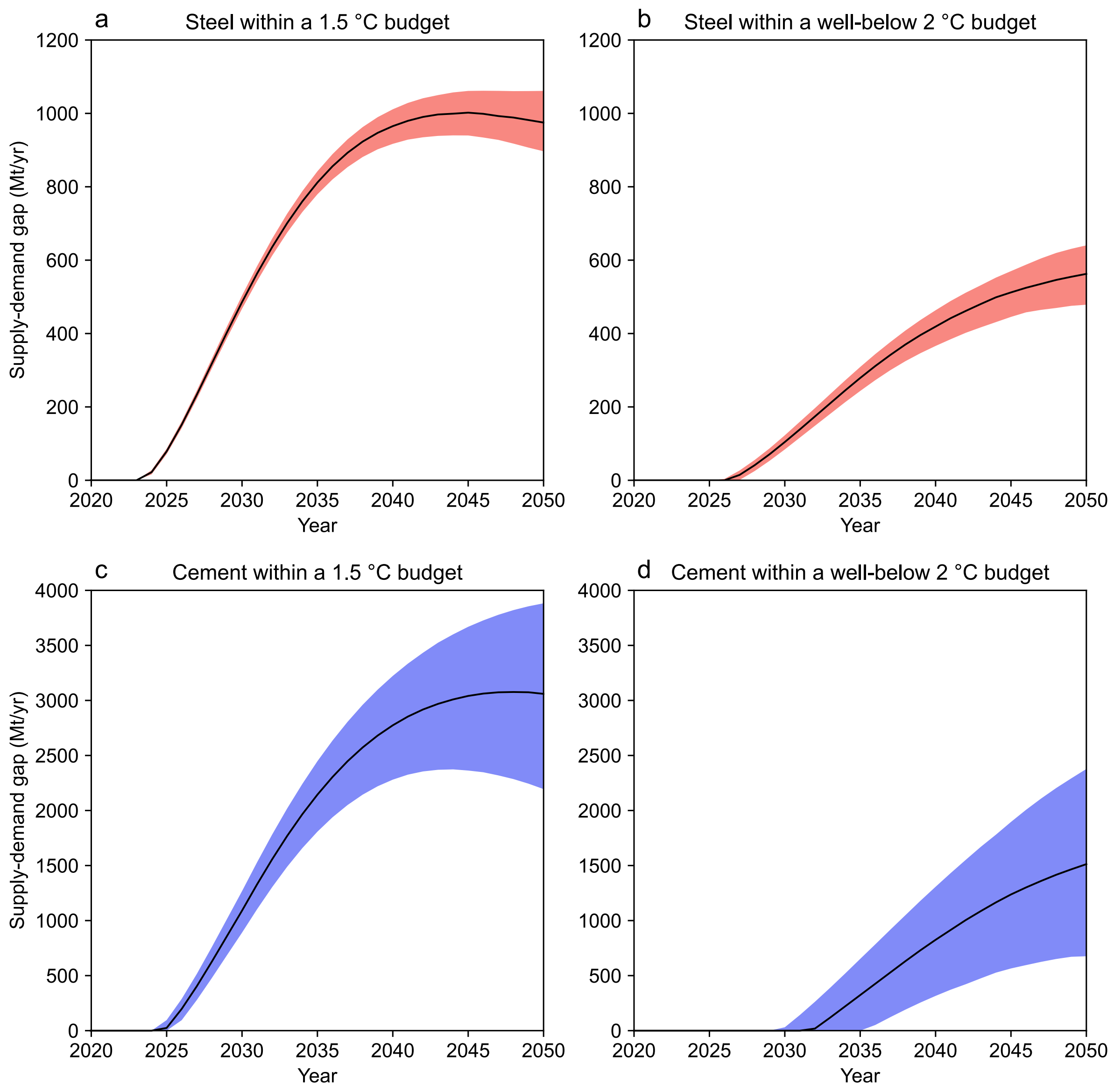


**Supplementary Fig. 9** Gap between feasible supply and expected baseline demand. (a) Steel sector within a 1.5 °C budget. (b) Steel within a well-below 2 °C budget. (c) Cement within a 1.5 °C budget. (d) Cement within a well-below 2 °C budget. The solid black line shows the median, and the colored bands around it show the interquartile range. We assume that the gap between feasible supply and expected demand for steel and cement will be filled by the current emission-intensive production processes (i.e., BF-BOF). This would entail carbon emissions of approximately 2.1 t-$CO_2$/t-steel or 0.6 t-$CO_2$/t-cement. The consequences of such emissions would be severe, potentially resulting in cumulative emissions of up to ~160 Gt-$CO_2$ by 2050. This amount represents ~40% of the remaining 1.5°C budget or ~20% of the remaining well-below 2°C budget.

**Supplementary Table 5** Material saving potential through a range of material efficiency strategies in the construction sectors. Potential savings are multiplicative rather than additive across the strategies. Estimates are based on weighted averages for each use. Data are based on Refs [30–38].

| Strategy | Description | Buildings | Infrastructure | Construction sectors |
|---|---|---|---|---|
| Efficient design | Reduced overdesign, optimized design | 20-50% | 20-30% | 20-45% |
| Fabrication yield improvement | Prefabrication, better specification, use of over-ordered materials for other purposes | 1-2% | 1-2% | 1-2% |
| More intense use | Smaller residences, larger household sizes, fewer second homes, dual-use spaces, shared or multi-purpose office spaces, centralization of urban functions | 20-50% | 0-10% | 15-35% |
| Lifetime extension | Proactive maintenance, repurposing, reuse, remanufacturing, changing consumer preferences | 10-20% | 0-5% | 5-15% |
| Total | | 40-80% | 20-40% | 35-70% |

**Supplementary Table 6** Material saving potential through a range of material efficiency strategies in the manufacturing sectors. Potential savings are multiplicative rather than additive across the strategies. Data are based on Refs [30,32,39–44].

| Strategy | Description | Vehicles | Machinery | Consumer goods | Manufacturing sectors |
|---|---|---|---|---|---|
| Efficient design | Light-weighting, right-sizing | 25-45% | 30% | 30% | 30-35% |
| Fabrication yield improvement | Improved blanking and stamping process, reduced material overlap, nested parts design | 5-10% | 5% | 10% | 5-10% |
| More intense use | Car-sharing, ride-sharing, better scheduling and coordination | 25-40% | 10% | 0% | 10-15% |
| Lifetime extension | Reuse, remanufacturing, changing consumer preferences, revised inspection system | 10-20% | 10% | 50% | 25-30% |
| Total | | 50-75% | 45% | 70% | 55-65% |

**Supplementary References**


1. IEA. *Energy Technology Perspectives 2010*. (OECD, 2010). doi:10.1787/energy_tech-2010-en.
2. IEA. *Energy Technology Perspectives 2012*. (OECD, 2012). doi:10.1787/energy_tech-2012-en.
3. IEA. *Energy Technology Perspectives 2014*. (OECD, 2014). doi:10.1787/energy_tech-2014-en.
4. IEA. *Energy Technology Perspectives 2015*. (OECD, 2015). doi:10.1787/energy_tech-2015-en.
5. IEA. *Energy Technology Perspectives 2016*. (OECD, 2016). doi:10.1787/energy_tech-2016-en.
6. IEA. *Energy Technology Perspectives 2017*. (OECD, 2017). doi:10.1787/energy_tech-2017-en.
7. IEA. *Energy Technology Perspectives 2020*. (OECD, 2020). doi:10.1787/d07136f0-en.
8. IEA. *Energy Technology Perspectives 2023*. (OECD, 2023).
9. IEA. *Net Zero by 2050*. (OECD, 2021). doi:10.1787/c8328405-en.
10. Pauliuk, S., Milford, R. L., Müller, D. B. & Allwood, J. M. The steel scrap age. *Environ Sci Technol* **47**, 3448–3454 (2013).
11. Cullen, J. M., Allwood, J. M. & Bambach, M. D. Mapping the global flow of steel: From steelmaking to end-use goods. *Environ Sci Technol* **46**, 13048–13055 (2012).
12. Watari, T., Hata, S., Nakajima, K. & Nansai, K. Limited quantity and quality of steel supply in a zero-emission future. *Nat Sustain* (2023) doi:10.1038/s41893-022-01025-0.
13. Allwood, J. M., Dunant, C. F., Lupton, R. C. & Serrenho, A. C. H. Steel Arising. 1–20 (2019) doi:10.17863/CAM.40835.
14. Müller, D. B. *et al.* Carbon emissions of infrastructure development. *Environ Sci Technol* **47**, 11739–11746 (2013).
15. Cao, Z., Shen, L., Løvik, A. N., Müller, D. B. & Liu, G. Elaborating the History of Our Cementing Societies: An in-Use Stock Perspective. *Environ Sci Technol* **51**, 11468–11475 (2017).
16. Ryan, N. A., Miller, S. A., Skerlos, S. J. & Cooper, D. R. Reducing CO 2 Emissions from U.S. Steel Consumption by 70% by 2050. *Environ Sci Technol* (2020) doi:10.1021/acs.est.0c04321.
17. Fan, Z. & Friedmann, S. J. Low-carbon production of iron and steel: Technology options, economic assessment, and policy. *Joule* vol. 5 829–862 Preprint at https://doi.org/10.1016/j.joule.2021.02.018 (2021).
18. Gast, L., Cabrera Serrenho, A. & Allwood, J. M. What Contribution Could Industrial Symbiosis Make to Mitigating Industrial Greenhouse Gas (GHG) Emissions in Bulk Material Production? *Environ Sci Technol* **56**, 10269–10278 (2022).
19. Bhaskar, A., Assadi, M. & Somehsaraei, H. N. Decarbonization of the iron and steel industry with direct reduction of iron ore with green hydrogen. *Energies (Basel)* **13**, (2020).
20. Vogl, V., Åhman, M. & Nilsson, L. J. Assessment of hydrogen direct reduction for fossil-free steelmaking. *J Clean Prod* **203**, 736–745 (2018).
21. Global Cement and Concrete Association. GNR Project. https://gccassociation.org/sustainability-innovation/gnr-gcca-in-numbers/ (2021).

22. International Energy Agency. *Technology Roadmap: Low-Carbon Transition in the Cement Industry*. (2018).
23. Watari, T., Cao, Z., Serrenho, A. C. & Cullen, J. Growing role of concrete in sand and climate crises. *iScience* **26**, 106782 (2023).
24. Perpiñán, J. *et al.* Integration of carbon capture technologies in blast furnace based steel making: A comprehensive and systematic review. *Fuel* **336**, (2023).
25. Voldsund, M. *et al.* Comparison of technologies for CO 2 capture from cement production—Part 1: Technical evaluation. *Energies (Basel)* **12**, (2019).
26. IPCC. *Climate Change 2021: The Physical Science Basis. Contribution of Working Group I to the Sixth Assessment Report of the Intergovernmental Panel on Climate Change*. (2021).
27. Calverley, D. & Anderson, K. *Phaseout Pathways for Fossil Fuel Production Within Paris-compliant Carbon Budgets*. http://man.ac.uk/04Y6Bo] (2022).
28. Raupach, M. R. *et al.* Sharing a quota on cumulative carbon emissions. *Nat Clim Chang* **4**, 873–879 (2014).
29. Pye, S. *et al.* Regional uptake of direct reduction iron production using hydrogen under climate policy. *Energy and Climate Change* 100087 (2022) doi:10.1016/j.egycc.2022.100087.
30. Milford, R. L., Pauliuk, S., Allwood, J. M. & Müller, D. B. The Roles of Energy and Material Efficiency in Meeting Steel Industry CO2 Targets. *Environ Sci Technol* **47**, 3455–3462 (2013).
31. Shanks, W. *et al.* How much cement can we do without? Lessons from cement material flows in the UK. *Resour Conserv Recycl* **141**, 441–454 (2019).
32. IEA. *Material efficiency in clean energy transitions*. (2019) doi:10.1787/aeaaccd8-en.
33. Dunant, C. F., Drewniok, M. P., Orr, J. J. & Allwood, J. M. Good early stage design decisions can halve embodied CO2 and lower structural frames' cost. *Structures* **33**, 343–354 (2021).
34. Watanabe, S. I. *et al. Material Efficiency as a Means to Lower Environmental Impacts from Concrete*. (2021) doi:10.7922/G2RJ4GS8.
35. Pamenter, S. & Myers, R. J. Decarbonizing the cementitious materials cycle: A whole-systems review of measures to decarbonize the cement supply chain in the UK and European contexts. *J Ind Ecol* **25**, 359–376 (2021).
36. Grubler, A. *et al.* A low energy demand scenario for meeting the 1.5 °c target and sustainable development goals without negative emission technologies. *Nat Energy* **3**, 515–527 (2018).
37. Watari, T., Cao, Z., Hata, S. & Nansai, K. Efficient use of cement and concrete to reduce reliance on supply-side technologies for net-zero emissions. *Nat Commun* **13**, (2022).
38. Pauliuk, S. & Müller, D. B. The role of in-use stocks in the social metabolism and in climate change mitigation. *Global Environmental Change* **24**, 132–142 (2014).
39. Horton, P. M., Allwood, J. M. & Cleaver, C. Implementing material efficiency in practice: A case study to improve the material utilisation of automotive sheet metal components. *Resour Conserv Recycl* **145**, 49–66 (2019).
40. Wolfram, P., Tu, Q., Heeren, N., Pauliuk, S. & Hertwich, E. G. Material efficiency and climate change mitigation of passenger vehicles. *J Ind Ecol* 1–17 (2020) doi:10.1111/jiec.13067.
41. Nakamoto, Y. & Kagawa, S. Role of vehicle inspection policy in climate mitigation: The case of Japan. *J Environ Manage* **224**, 87–96 (2018).

42. Pauliuk, S., Heeren, N., Berrill, P., Fishman, T. & Hertwich, E. G. Global scenarios of resource and emission savings from material efficiency in residential buildings and cars. *Nat Commun* **12**, 5097 (2021).
43. Watari, T., Nansai, K., Nakajima, K. & Giurco, D. Sustainable energy transitions require enhanced resource governance. *J Clean Prod* **312**, 127698 (2021).
44. Klose, S. & Pauliuk, S. Quantifying longevity and circularity of copper for different resource efficiency policies at the material and product levels. *J Ind Ecol* 1–15 (2021) doi:10.1111/jiec.13092.
